# The ionophore resistance genes *narA* and *narB* are geographically widespread and linked to resistance to medically important antibiotics

**DOI:** 10.1128/msphere.00243-25

**Published:** 2025-06-17

**Authors:** Asalia Ibrahim, Jason Au, Alex Wong

**Affiliations:** 1 Department of Biology, Carleton University 120895, Ottawa, Ontario, Canada; Hackensack Meridian Health Center for Discovery and Innovation, Nutley, New Jersey, USA

**Keywords:** antimicrobial resistance, ionophore, One Health, agriculture

## Abstract

Ionophores are a class of antibiotics used widely in animal production as anti-coccidials and for growth promotion. Since ionophores are not used in human medicine, it has largely been assumed that they do not contribute to medically important antimicrobial resistance (AMR). Nonetheless, there is increasing concern that ionophore usage could co-select for clinically relevant AMR, since the ionophore resistance genes *narA* and *narB* have been found in linkage with multiple AMR genes. We investigated the global distribution and AMR linkage of *narA* and *narB* using publicly available data. These ionophore resistance genes can be found worldwide, with >2,400 *narAB*-bearing isolates reported from 51 countries. Isolates were derived from a range of host species, including poultry, cattle, and humans. *narAB* was linked with an average of over 10 resistance determinants for AMR, including many medically important antibiotics. These observations indicate that we cannot assume that ionophore use is risk-free, with clear potential for co-selection for clinically relevant AMR.

**IMPORTANCE:**

Ionophores are a type of antibiotic used to promote growth in cattle and pigs and to treat parasitic infections in poultry. It has been assumed that ionophore use in animals does not pose a risk for humans. However, growing evidence suggests that ionophore use may select for medically relevant antibiotic resistance. Using analyses of public data, we found that ionophore resistance is widespread and that it is usually linked to resistance genes for medically relevant drugs. There is thus clear potential for ionophore use to impact the presence of antibiotic resistance genes in the food supply.

## OBSERVATION

Ionophores are a class of antibiotics used widely in animal production. They have two primary uses: as anti-coccidials in poultry (1) and for growth promotion in swine and cattle (e.g., reference 2). Ionophores are widely used, representing 37% of antibiotics used in food-producing animals in the United States in 2022, with almost 4.2 million kilograms sold (3).

Ionophores are not used in human medicine due to toxicity. As such, it has generally been assumed that ionophores do not contribute to the burden of antimicrobial resistance (AMR) in human populations. Their use is thus regulated less strictly than drugs that are used in humans or drugs belonging to classes that are used in humans. Canadian and US regulations, for example, classify ionophores as having low or no importance for human medicine (4, 5).

However, recent evidence suggests that ionophore use in animal populations has the potential to contribute indirectly to human-relevant AMR (reviewed in references 6–8). Indirect selection for human-relevant AMR could arise in two ways (8): (i) cross-resistance, whereby a gene that causes ionophore resistance also causes resistance to a medically important antibiotic (MIA), or (ii) co-selection, whereby a gene that causes ionophore resistance is genetically linked to genes causing resistance to MIAs. It has been argued that cross-resistance between ionophores and MIAs is unlikely, since ionophores, as polyether antibiotics, do not belong to any drug class that is used in humans (e.g., reference 9).

In the last few years, several studies have raised the possibility of co-selection between agriculturally used ionophores and MIAs. All of these studies focus on NarAB, a transporter that confers resistance to narasin, salinomycin, and maduramicin in *Enterococcus faecium* (10). The *narA* and *narB* genes were initially found on the same plasmid as vancomycin resistance genes in *E. faecium* isolates from Swedish broilers (11). More recently, *narAB* was found on plasmids with genes causing resistance to erythromycin and tetracycline, from isolates in the Netherlands (12). Linkage between known ionophore resistance genes and genes causing resistance to MIAs indicates that ionophore use has the potential to indirectly contribute to the burden of human-relevant AMR. Consistent with this hypothesis, cessation of narasin use by the Norwegian broiler industry in 2016 corresponded with a reduction in the prevalence of vancomycin-resistant *Enterococci* (13).

Despite the possibility for indirect selection, actual human risks posed by ionophore use in animals are still unclear. It is unknown, for example, whether the *narAB* genes are geographically widespread, nor whether they are consistently linked with resistance to MIAs. We address these questions using publicly available data.

### The *narAB* operon is geographically widespread

We investigated the geographic distribution of the narasin resistance genes *narA* and *narB*. We searched the NCBI Pathogens database, which harbours genome sequence data from pathogens recovered during surveillance and research, for all genomes annotated as harboring *narA*. We then refined this list to include only isolates with complete copies of both *narA* and *narB* (as predicted in the Pathogens database), yielding 2,442 isolates. We extracted from the Pathogens data set the following information: country of origin, host bacterial species, host organism (if indicated), and AMR annotations (predicted by NCBI using AMRFinderPlus [14]).

**Fig 1:**
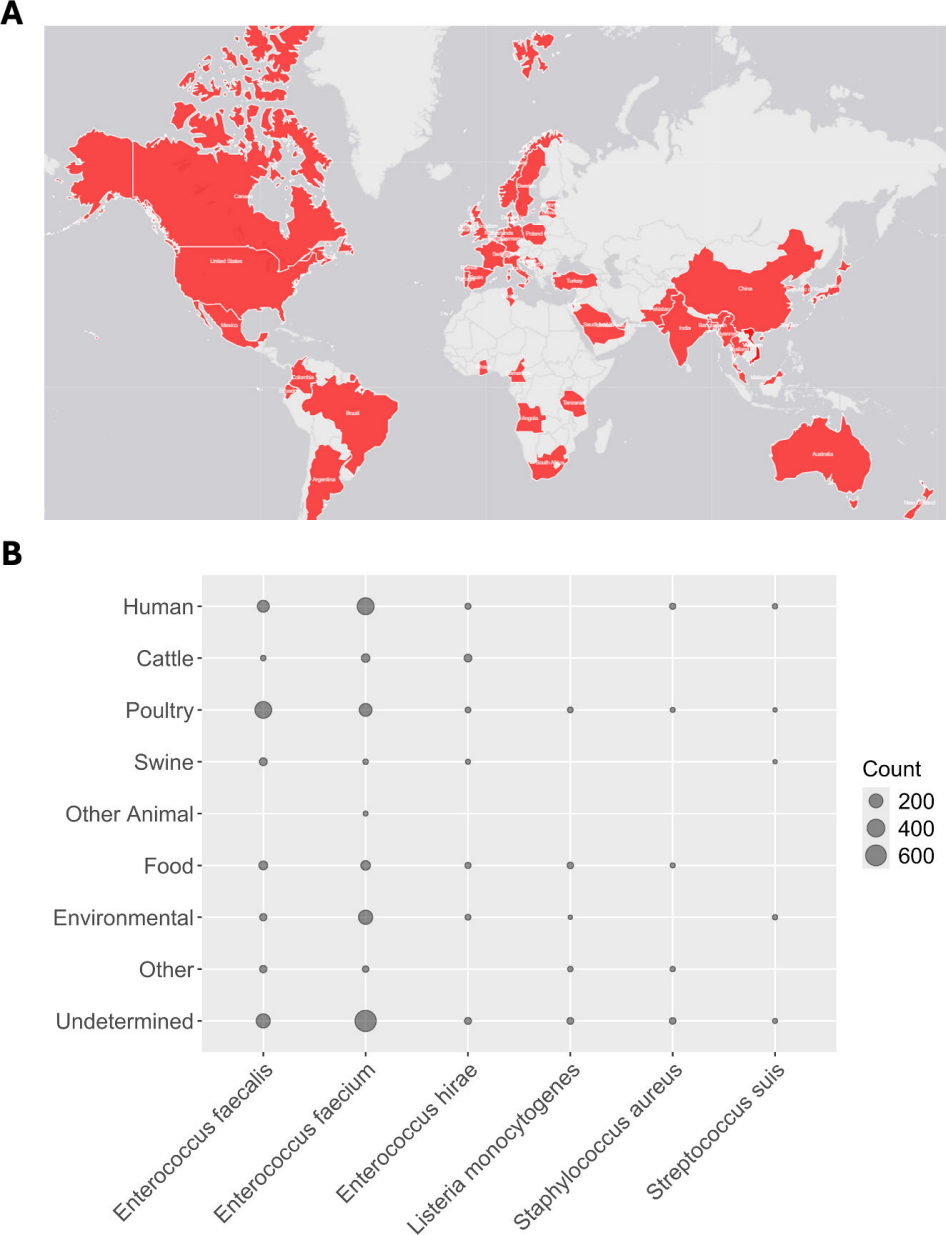
Distribution of *narA/B*. (A) World map showing the geographic distribution of *narAB*. (B) Distribution of *narAB* amongst bacterial and host species. Bubble size is proportional to the number of detected isolates. Four bacterial species are excluded due to total sample sizes of less than 5 (*Campylobacter jejuni*, *Clostridium perfringens*, *Listeria innocua,* and *Streptococcus agalactiae*).

*narAB* was originally isolated in *E. faecium* from Swedish broiler chickens (15), and a recent report identified additional isolates in the Netherlands (12). Our investigation indicates that *narAB* is found worldwide, with 2442 isolates from 51 countries spanning every continent except Antarctica (Fig. 1A). We note that sampling heterogeneity precludes comparison of the frequency of *narAB* between countries, so we present only presence/absence data. *narAB* sequences were identified in 10 bacterial species, with *Enterococcus faecalis* and *E. faecium* most highly represented. *narAB*-bearing isolates were derived from a range of hosts, including poultry, swine, and cattle (Fig. 1B). Notably, over 500 isolates were derived from humans, indicating that *narAB* is not limited to non-human animals as hosts.

### *narAB* is linked to human relevant AMR genes

**Fig 2:**
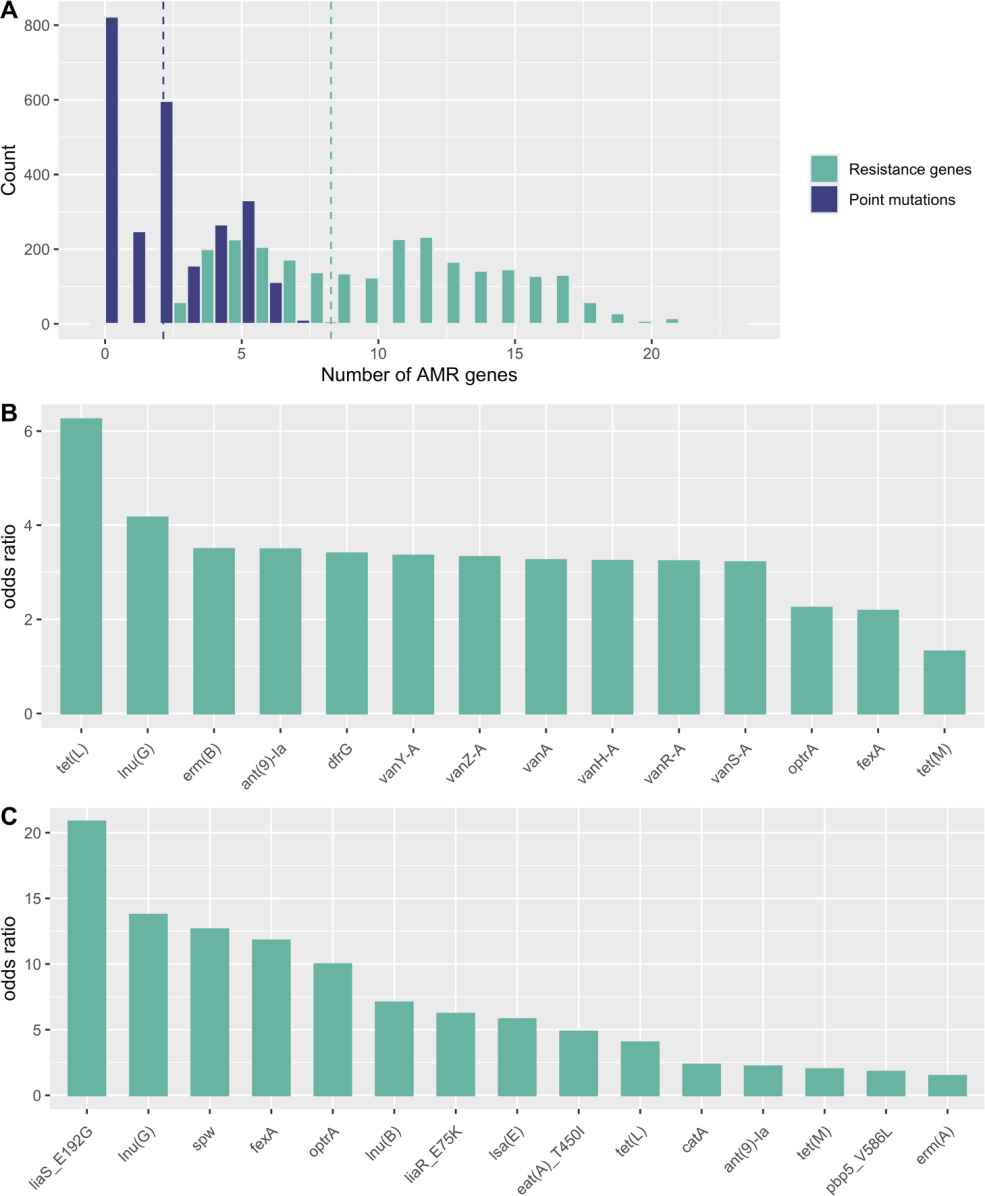
AMR determinants in *narAB*-bearing isolates. (**A**) Histogram of the number of AMR genes (green) or point mutations (blue) present in *narAB* isolates, not including *narAB*. Dashed lines indicate the means for the two groups. (**B, C**) Barplots showing statistical association between *narAB* and human-relevant AMR determinants in *E. faecalis* (**B**) and *E. faecium* (**C**). For each gene or mutation, Fisher’s exact test odds ratio is given and represents the fold increase in odds of observing the given gene (mutation) in an isolate carrying *narAB*; *P* < 0.001 in all cases shown here.

Previous studies have reported a linkage between *narAB* and resistance genes for vancomycin (10, 15) or for erythromycin and tetracycline (12). We note that co-selection requires genetic linkage between different resistance genes and can arise from either physical linkage (e.g., genes on the same plasmid) or the presence in the same genome in a largely clonal species (8). The >2,400 *narAB*-containing isolates examined here harbored an average of 8.26 resistance genes and 2.13 point mutations conferring resistance (Fig. 2A). Resistance to medically important antimicrobials was widespread; the most common resistance genes detected are predicted to confer resistance to erythromycin (*ermB*), tetracycline [*tet*(*M*)], or aminoglycosides [*aac(6′)-I*]. The most common point mutations are predicted to confer daptomycin resistance (*liaR* E75K), ampicillin resistance (*pbp5* E629V), or the multidrug-resistant LSAP phenotype [*eat*(*A*) T450I (16)].

We further investigated the possibility of co-selection between *narAB* and resistance to MIAs by estimating statistical associations between resistance determinants in *E. faecalis* and *E. faecium* (Fig. 2B and C). In *E. faecalis,* we found positive associations (Fisher’s exact test odds ratio > 1, *P* < 0.001) between *narAB* and 13 resistance genes, including all members of the *vanA* gene cluster. In *E. faecium*, we found positive associations between *narA/B* and 11 resistance genes, as well as 4 point mutations.

Linkage between narasin resistance and vancomycin resistance was first reported in Swedish isolates of *E. faecium* (11, 15). It is now clear that *narAB* is distributed worldwide (Fig. 1A) and is found in linkage with a wide range of resistance genes for medically important antibiotics (Fig. 2). *narAB* can be found in a number of host species, including humans. While the direction of *narAB* transfer has not been established, transfer from farm animals to humans seems most likely. These observations indicate that we cannot assume that ionophore use is risk-free, with clear potential for co-selection for clinically relevant AMR.
